# Boron Nitride Nanotubes Assist the Self-Assembly of Spherical Cholesteric Liquid Crystal Shells of Cellulose Nanocrystals in Water

**DOI:** 10.1021/acs.langmuir.6c00394

**Published:** 2026-05-04

**Authors:** Tanner L. Larson, Brandon J. Heppe, Benjamin S. Flavel, Ralph Krupke, Geyou Ao

**Affiliations:** † Department of Chemical and Biomedical Engineering, Washkewicz College of Engineering, 2564 Cleveland State University, 2121 Euclid Avenue, Cleveland, Ohio 44115, United States; ‡ Institute of Nanotechnology, 150232 Karlsruhe Institute of Technology, 76131 Karlsruhe, Germany; § Department of Materials Science, Technical University of Darmstadt, 64287 Darmstadt, Germany; ∥ Institute of Quantum Materials and Technologies, 150232 Karlsruhe Institute of Technology, 76131 Karlsruhe, Germany

## Abstract

Spherical confinement of cholesteric liquid crystal (ChLC) droplets is emerging as an intriguing approach for achieving complex ordered structures for photonic applications and beyond. Previously, this has been achieved primarily through microfluidic assembly utilizing immiscible solvents or immiscible mesogen–solvent systems, such as oil–water or thermotropic liquid crystal–water systems, respectively. Here, we show the spontaneous assembly of spherical ChLC droplets with isotropic cores from lyotropic liquid crystals of mixed nanorods containing boron nitride nanotubes (BNNTs) and cellulose nanocrystals (CNCs) in all-aqueous environments. We show that the mixing of as low as 0.094 vol % of pectin-coated BNNTs with isotropic dispersions of 3.20 ± 0.04 vol % of CNCs enables the self-assembly of spherical ChLC droplets of micrometer size in water. This is potentially caused by the larger-aspect-ratio BNNTs modulating the overall nanorod alignment within the cholesteric shell of the resulting liquid crystal-in-water system of BNNT/CNC mixtures. Specifically, the size and negative charge of pectin-coated BNNTs are compatible with the negatively charged CNC template. By slightly increasing the BNNT concentration up to 0.121 vol %, we observed the thickness of ChLC shells to grow by roughly 53%, suggesting a synergistic liquid crystal phase behavior in BNNT/CNC mixtures. These findings provide important insights into the scalable, water-based self-assembly of mixed nanorod systems with integrated properties in a spherical confinement, broadening applications for photonics, biochemical sensing, optical films, and nanomaterial templating.

## Graphical abstract

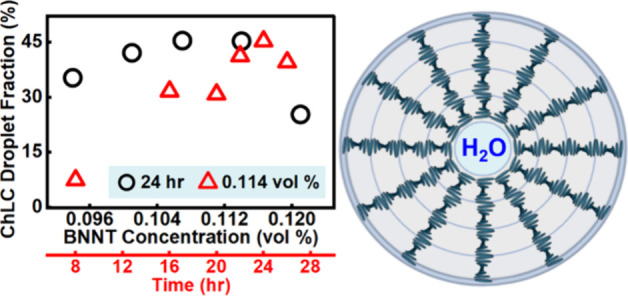

## Introduction

Lyotropic liquid crystals of rodlike nanomaterials are ideal precursors for the macroscopic assembly of diverse, ordered structures for applications, including photonics, biochemical sensors, aligned films and fibers, and material templating. Bulk liquid crystals of millimeter- and larger-sized polydomains that are formed from dispersions of individualized nanorods in a solvent have been explored extensively, where property enhancements are achieved through long-range ordering of nanoscale building blocks. This leads to the maximum translation of inherent nanoscale properties to macroscopic objects that are processed at industrially relevant production speeds, which is exemplified by lyotropic nematic liquid crystals of quasi-one-dimensional carbon nanotubes (CNTs).^1–3^ In comparison, curved confinement of nanorods, typically in micron-sized spherical geometries, which exhibits cholesteric phases and tunable topological defects for applications such as molecular templating and micro-optics development, remains largely unexplored.^4,5^

Recent advances have demonstrated the microfluidic-mediated, spherical confinement of rodlike cellulose nanocrystals (CNCs) into cholesteric liquid crystal (ChLC) shells.^6,7^ The helical order of these ChLC shells emanates from the immiscible water/oil (i.e., liquid crystal droplet/oil) interface, accompanied by a requisite isotropic droplet core (i.e., the radial point defect, or hedgehog) to mitigate the significant increase in the elastic energy approaching the droplet center at the equilibrium state. The isotropic droplet core can be utilized to encapsulate various molecules and nanoparticles, in order to further tune the optical properties and functions of ChLC shells.^6,7^ Structurally, CNCs are aligned tangentially at the droplet–oil interface with a radial orientation of the helical axis, affording a defined pitch size within the shell. This type of CNC ordering in spherical confinement translates to the intrinsic optical property of ChLC droplets, which is to selectively reflect circularly polarized light of defined wavelengths and handedness in an omnidirectional fashion.^8–11^ Specifically, ChLC shells that are formed from lyotropic nanorods of CNCs possess a micron-sized pitch, which correlates to light reflection in the infrared optical window based on Bragg’s law, offering unique applications in specialty and security coatings that are invisible to human eyes.^9^ Bulk cholesteric droplets of CNCs without core–shell structures were also reported, where a thermodynamically incompatible polymer aqueous two-phase system was utilized to obtain an emulsion of liquid crystal droplets-in-liquid crystal medium with global cholesteric alignment.^12^ Compared to lyotropic ChLCs in spherical confinement, many studies have focused on the microfluidic assembly of short-pitch ChLC shells and droplets using water-immiscible, small molecules of thermotropic liquid crystals, where several hundred nanometers of pitch sizes generally yield visible light reflection.^5,8,10,11^ Regardless of these recent efforts, the self-assembly of spherical ChLC shells of micrometer scale from lyotropic nanorods in an all-aqueous environment, an ideal green solvent, has not been achieved. This is due to the inherent limitation of vanishingly small interfacial tension in water-in-water emulsions, which is typically of the order of 10^–7^ to 10^–6^ N m^–1^ (i.e., ≈10^4^ to 10^5^ times smaller than that of typical oil–water emulsions) at the interface of isotropic and liquid crystal phases in aqueous suspensions of filamentous colloids, including CNCs.^13,14^ This leads to the formation of typical metastable, elongated liquid crystal tactoids in the biphasic regime for aqueous dispersions of rodlike colloids, rather than spherical droplets or shells.^13,14^

CNCs are renewable, hydrophilic (i.e., due to inherent −OH groups), and negatively charged nanorods, due to their sulfate half-ester groups that are introduced during the commonly used process of sulfuric acid hydrolysis.^15^ The lyotropic ChLC phase behavior of CNCs in water has been studied extensively. These include their morphological versatility in forming bulk liquid crystals^16–18^ and confined liquid crystals of spherical and cylindrical shapes,^6,7,19^ as well as their structural templating of other nanomaterials to produce ordered composite materials.^20,21^ Particularly, nanomaterials of compatible size and surface charge can be templated within the liquid crystal matrix of CNCs, which possess roughly 25–50 nm of average spacing between negatively charged CNC nanorods.^22^ In comparison, boron nitride nanotubes (BNNTs) are synthetic and hydrophobic nanorods of high aspect ratios with promising applications in electronics, aerospace, and thermal management materials, due to their unique physiochemical properties,^23–25^ neutron shielding,^26^ and thermal and chemical stabilities.^27^ Stable dispersion of BNNTs in water via electrostatic and steric repulsions can be accomplished by noncovalent complexation with various dispersants, such as surfactants,^28–30^ polymers,^29,31^ and biomolecules (e.g., DNA^32–34^ and saccharides^35,36^ ), through van der Waals, π–π stacking, and hydrophobic interactions with the nanotube surfaces. Moreover, the anisotropic structure and stiffness of BNNTs, satisfying two prerequisites of molecular mesogens to form liquid crystals, have led to the formation of lyotropic liquid crystals, although they have been bulk nematic phases only (i.e., long-range order without the helical orientation).^26,37–39^ Producing ordered optical materials with unique structures and integrated properties of nanoscale building blocks will further realize nanomaterial applications in optoelectronics and photonics beyond aligned films and fibers^30,33,37,38^ and composite materials.^20,21^

Here, we demonstrate the spontaneous assembly of micrometer-sized, spherical ChLC droplets with an isotropic core in water by mixing small amounts (i.e., ≈0.020–0.177 mass %) of individually dispersed pectin-coated BNNTs (pectin-BNNTs) and isotropic CNC dispersions of 5.14 ± 0.06 mass %. These ChLC shells exhibit the characteristic features of a spherical cholesteric droplet (i.e., a Maltese cross and alternating bright and dark concentric rings) under polarized optical light microscopy (POM). Additionally, we identified key factors for controlling the formation of ChLC shells, including the BNNT concentration, sample mixing conditions that promote nanorod interactions, and time needed for ChLC shells to evolve to the equilibrium state.

## Experimental Section

### Dispersions of BNNTs and CNCs

Dispersions of the few-walled BNNT material (refined puffball, lot no: #Y2B01210503E, <1 mass % elemental boron, BNNT Materials) in deionized (DI) water were produced using pectin (from citrus peel, MW ≈ 10–300 kDa, galacturonic acid ≥74.0% (dried basis), Sigma-Aldrich). Specifically, mixtures of BNNTs:pectin = 1:2 by mass at a total volume of 4 mL and a starting BNNT concentration of 4 mg mL^–1^ were bath sonicated for 1 h at room temperature, followed by probe tip ultrasonication (VCX 130, Sonics and Materials Inc.) in an ice bath for 1 h at a power level of 8 W (i.e., corresponding to 45% amplitude) using a 2 mm diameter probe. The supernatant was collected after centrifugation at 5000*g* for 30 min at 19 °C and used as the stock sample, unless indicated otherwise. These optimum dispersion conditions were determined by testing varying relative centrifugal forces from 1000 to 17,000*g* (Figure S1) and mass ratios of BNNTs:pectin from 1:1 to 1:8 (Figure S2).

Aqueous dispersions of CNCs (CNC-HS-FD, high-sulfonic group content, freeze-dried, Cellulose Lab) at varying concentrations (i.e., 5–13 mass %) were obtained by probe tip ultrasonication of the 4 mL sample for 1 h at a power level of 8 W in an ice bath. Tip sonication was split into two 30 min intervals, and between the intervals, the ice bath was refilled, and the sample was vortex mixed before continuing the sonication process. Here, all percentages specified for chemical concentrations are reported on a mass basis unless indicated otherwise.

The conversion of BNNT and CNC concentrations from mass % to volume fraction, ϕ, were determined by taking the average of previously reported density values of 1.50 g cm^–3^ for BNNTs,^40,41^ 1.64 g cm^–3^ for CNC,^16,42^ and 1.60 g cm^–3^ for pectin^43,44^ (Table S1).

### Phase Behavior of CNC Dispersions

Aqueous dispersions of CNCs at 1 mL volume and varying concentrations of 5–13% in sealed glass vials were vortex mixed for 90 s and then placed on an orbital platform shaker (Grant Bio PSU-10i, Grant Instruments) at 300 rpm with 1 cm orbital diameter for 24 h at ambient conditions before characterization.

### Self-Assembly of ChLC Shells from BNNT/CNC Mixtures

Typically, BNNT/CNC mixtures were prepared by mixing 750 μL of varying concentrations of pectin-coated BNNT dispersions and 730–760 μL of 10% CNC dispersion in 20 mL glass vials, where the liquid distribution in each vial on an orbital shaker corresponds to sample thicknesses of roughly 3.5–8.0 mm. The CNC dispersion was added at slightly varying volumes to ensure a constant mass ratio of pectin-BNNTs:CNCs across all of the samples. The resulting BNNT/CNC mixtures have a final BNNT concentration in the range of 0.020–0.177% (i.e., ϕ_BNNTs_ = 0.013–0.121 vol %), while keeping concentrations of CNCs and unbound, free pectin constant at 5.14 ± 0.06% (i.e., ϕ_CNC_ = 3.20 ± 0.04 vol %) and 0.29 ± 0.04% (i.e., ϕ_pectin_ = 0.18 ± 0.03 vol % and 3.03 ± 0.43 mg/mL), respectively. Glass vials containing various BNNT/CNC mixtures were sealed and vortex mixed for 90 s before being placed on an orbital platform shaker at 300 rpm with 1 cm orbital diameter for various time periods (i.e., 30 min to 5 days) at ambient conditions. Stationary samples without continuous mixing on an orbital platform shaker were used as controls.

### Optical Spectroscopy Characterization

UV–vis absorbance measurements of BNNT samples were performed using a Jasco V-760 spectrophotometer over the wavelength range of 187–800 nm using a quartz cuvette of 10 mm path length. The concentration of individually dispersed pectin-BNNT complexes was determined using a BNNT extinction coefficient of 178.54 mL mg^–1^ cm^–1^ at 205 nm by deconvoluting the absorption peak of BNNTs by peak fitting using a multiple linear regression model (Figures S3 and S4). The amount of excess free pectin was determined using the pectin extinction coefficient of 1.13 mL mg^–1^ cm^–1^ at 220 nm wavelength (Figure S5).

### ATR-FTIR Spectroscopy

Attenuated total reflectance Fourier transform infrared (ATR-FTIR) measurements were obtained using a PerkinElmer Spectrum Two FT-IR spectrometer with a resolution of 0.5 cm^–1^ in the wavenumber range of 450–4000 cm^–1^. Supernatant samples of pectin solutions, which were ultrasonicated using conditions consistent with the preparation of pectin-BNNT dispersion, were lyophilized (FreeZone Freeze-Dryer, Labconco) for 24 h at 0.2 mbar and −50 °C for measurements. Our FTIR spectrum was baseline-corrected and fitted with Voigt profiles using Origin Pro 2025b (Figure S6). Specifically, the absorption bands at 1608 cm^–1^ correspond to the asymmetric stretching vibration of carboxylate anions (−COO^–^), as well as 1729 and 1747 cm^–1^ to the C=O stretching vibrations of carboxylic acids (−COOH) and carboxyl esters, respectively.^45^ The degree of esterification (DE) was determined by obtaining the ratio of the relative peak area for carboxyl esters over the sum of peak areas of the carboxylate anions, carboxylic acids, and carboxyl esters.^46^

### Optical Microscopy

Cross-polarized optical light microscopy (POM) and bright-field (BF) microscopy of various samples in transmission were performed on an Olympus BX51WI, B&B Microscope. The liquid crystal phase behavior of samples was characterized by POM after the 24 h mixing period, unless indicated otherwise. Samples were transferred on a microscope slide either using a 100 μL size pipet tip by cutting off the top part of the tip, leaving an opening of ≈1 mm diameter to minimize the potential shear effect, or using a spatula. Spherical ChLC shells were identified as droplets that have a uniform radius and display a Maltese cross, alternating bright and dark concentric rings, and an isotropic core. The pitch and size distributions of ChLC shells were measured from POM and BF images using ImageJ.

### Zeta Potential and pH Measurements

Zeta potential measurements of the pectin solution and aqueous dispersions of pectin-BNNTs and CNCs were performed using a Malvern Zetasizer Nano series Nano-ZS at room temperature, where samples were injected into a folded capillary zeta cell. The corresponding pH values of samples were measured using a Metter Toledo SevenCompact S220 pH/Ion meter at room temperature.

### Transmission Electron Microscopy

Images of CNC samples were collected using an FEI Tecnai T12 transmission electron microscope operating at 80 kV with a LaB6 filament and the Gatan 895 UltraScan 4k × 4k camera. Sample preparation was performed following a previously reported procedure.^47^ Briefly, a 10 μL droplet of 0.025 mg mL^–1^ CNC dispersion was deposited on a carbon Formvar-coated copper grid (300 mesh, Electron Microscopy Sciences) with glow discharge treatment, followed by incubation for 4 min. Excess sample was removed by gently blotting with filter paper (Vitrobot) and washed with DI water before applying 2% methylamine vanadate (VitroEase, Thermo Scientific) heavy metal stain for 4 min. The grid was then rinsed with DI water and allowed to dry for 1 h before imaging.

## Results and Discussion

We obtained stable dispersions of BNNTs in water by noncovalent complexation with the natural polysaccharide pectin, which is an effective dispersion agent for nanotubes, including the carbon counterparts of BNNTs–CNTs (Figures S1–S4).^35,48,49^ The resulting pectin-BNNTs showed a characteristic absorption peak of BNNTs at 205 nm (Figure S1). The estimated number-average length, *L*, of pectin-BNNTs is roughly 324 ± 133 nm based on our prior work utilizing the same sample dispersion method.^32^ The stock dispersions of pectin-BNNTs also contain an excess, unbound pectin of ≈6 mg mL^–1^. Based on molecular dynamics simulations, the stabilization of tubular nanostructures in water by various polysaccharides is driven mainly by van der Waals attraction and hydrophobic interactions between the carbohydrate pyranose rings and the hydrophobic surfaces of nanotubes.^48–50^ Adsorption of polysaccharides onto nanotube surfaces results in the formation of ordered, helical coatings around nanotubes with a certain degree of bare BNNT surfaces exposed to the environment, preventing their aggregation in water. Here, the zeta potential of pectin-BNNTs is found to be −40 mV, indicating a stable dispersion of nanotubes due to electrostatic stabilization in water (Table S2). Additionally, hydrophilic CNCs produced stable dispersions in water with a number-average length of 139 ± 48 nm, a width, *W*, of 6.1 ± 1.3 nm, and a zeta potential of −66 mV (Figure S7 and Table S2). The negative charges of both nanorods (i.e., CNCs and pectin-BNNTs) afford the CNC templating of BNNTs into ChLC shells with minimal aggregation through electrostatic repulsion of nanorods in water.^22^

When adding small amounts of pectin-BNNTs into isotropic CNC dispersions, we observed the spontaneous formation of micron-sized ChLC droplets with an isotropic core (i.e., topological defect) in an all-aqueous environment at equilibrium (i.e., typically at 20–24 h after mixing) (Figure 1). This is different from the ChLC shells reported previously for both lyotropic CNCs and thermotropic liquid crystals, which were assembled in an oil/water system only with microfluidics. Typically, our BNNT/CNC mixtures contain slightly varying BNNT concentrations (i.e., 0.094–0.121 vol %), while maintaining constant concentrations of 3.20 ± 0.04 vol % CNCs and 0.18 ± 0.03 vol % free pectin. CNCs are known to form a cholesteric phase in water, where nanorods are aligned locally along a unit vector—director ***n*** —while following a helical path about an axis perpendicular to the director. This cholesteric ordering of CNCs can be further utilized as a template to align nanoparticles that have a compatible size and surface charge. The average diameter of BNNT materials used here is roughly *D* ≈ 5.2 nm,^37,39^ which can fit within the average spacing between negatively charged, cholesteric CNCs (i.e., ≈25–50 nm)^22^ (Figure 1b). On POM, these ChLC shells exhibit the characteristic features a spherical cholesteric droplet, displaying a Maltese cross and alternating bright and dark concentric rings.^6^ This ordered structure corresponds to the tangential alignment of nanorods at the liquid crystal droplet/water interface with a radial orientation of the helical axis of the cholesteric phase.^6,7^ Representative BF and POM images of the self-assembled ChLC shells in BNNT/CNC mixtures containing ϕ_BNNTs_ = 0.107 vol % show sizes of 40.0 ± 8.4 μm for the droplet diameter, 11.6 ± 3.2 μm for the isotropic core diameter, and 8.4 ± 0.6 μm for the cholesteric pitch, which is determined by measuring a double distance between two adjacent concentric rings (Figure 1c–f).

**Figure 1:**
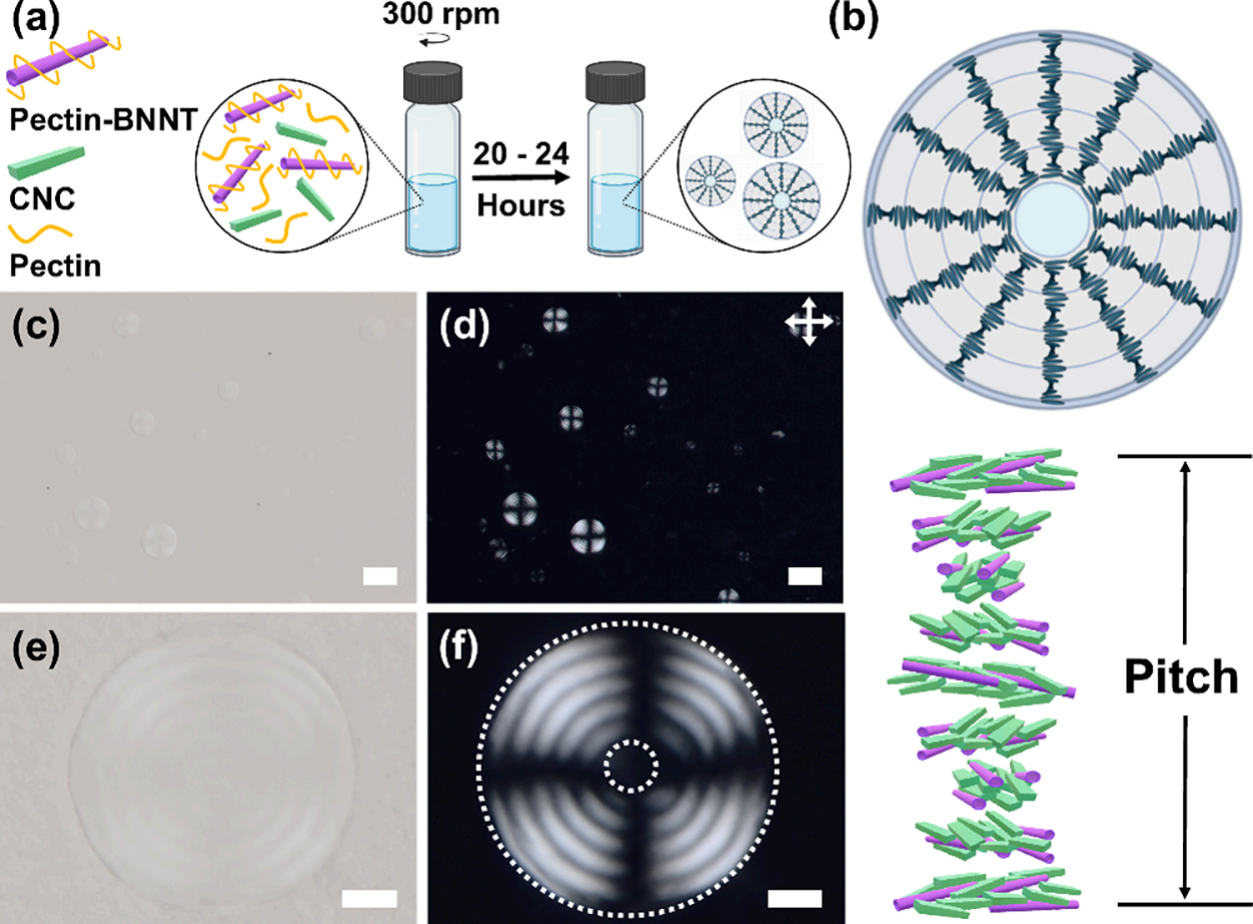
Self-assembly of spherical ChLC droplets with a liquid crystal shell and an isotropic core formed from BNNT/CNC mixtures in water. Schematics of (a) the assembly process of ChLC shells and (b) corresponding ordered structure of droplets. (c, e) BF optical microscopy and (d, f) corresponding POM images of ChLC droplets at ϕ_CNC_ = 3.24 vol % and ϕ_BNNTs_ = 0.107 vol % after 24 h mixing. Dotted circles in (f) correspond to the circumferences of the droplet and the isotropic core, respectively. Scale bars: (c, d) 50 and (e, f) 10 μm.

The spontaneous formation of spherical ChLC shells for our BNNT/CNC system in water demonstrates the unique role of mesogenic BNNTs in the aqueous CNC matrix. According to the Onsager theory, spontaneous alignment occurs in a lyotropic system of anisotropic mesogens, such as rods and platelets, with increasing concentration due to an increased overall entropy (i.e., resulting from an increase in translational entropy for alignment) and the restrictions on the degrees of freedom in the concentrated isotropic phase (i.e., related to a loss of rotational entropy).^51,52^ This leads to a critical concentration for the biphasic phase transition (i.e., ϕ_I_) to occur, at which liquid crystalline and isotropic phases coexist in equilibrium. Onsager found that this critical concentration is inversely proportional to the aspect ratio of mesogens with ϕ_I_ = 3.34/aspect ratio. While following the Onsager-like scaling, the critical concentrations of rodlike dispersions generally differ from the theoretically predicted values due to additional factors impacting the phase transition, such as sample polydispersity, molecular interactions, and solvent environments.^53,54^ Particularly, the ϕ_I_ of polydisperse samples, as demonstrated by various liquid crystals of nanorods, shifts to lower concentrations and forms a broader biphasic region, due to the presence of higher-aspect-ratio rods.^16,39,55,56^ For aqueous dispersions of CNCs only, we determined the critical concentration for phase transition from the concentrated isotropic to biphasic regime to be ϕ_I,CNC_ ≈ 3.43 vol % by POM, when small liquid crystal tactoids dispersed in a continuous isotropic phase first began to evolve (Figure S8). Above ϕ_I,CNC_, the liquid crystal domains of CNC samples continue to grow with increasing concentration and show birefringent microstructures with colors that change with rotation (Figure S8). Here, the aspect ratio of individually dispersed BNNTs (i.e., *L*/*D* = 62, *L* ≈ 324 nm, *D* ≈ 5.2 nm) is larger than that of CNCs (i.e., *L*/*W* = 23, *L* ≈ 139 nm, *W* ≈ 6.1 nm). Adding small amounts of larger-aspect-ratio, stiff BNNTs in isotropic dispersions of smaller-aspect-ratio CNCs enabled the formation of the ChLC phase in the resulting BNNT/CNC mixtures, where the CNC concentration in mixtures (i.e., ϕ_CNC_ = 3.20 ± 0.04 vol %) is lower than the estimated critical concentration for biphasic phase transition (ϕ_I,CNC_ ≈ 3.43 vol %) in lyotropic CNCs alone. Additionally, the total nanorod concentrations (i.e., ϕ_BNNTs+CNC_) in BNNT/CNC mixtures were less than ϕ_I,CNC_ (Table S1). The lowering of the overall nanorod concentration for the biphasic phase transition in BNNT/CNC mixtures by adding a small amount of larger-aspect-ratio BNNTs in the CNC matrix suggests a synergistic liquid crystal behavior of mixed, mesogenic nanorods of BNNTs and CNCs in water.

Moreover, ChLC droplets formed from BNNT/CNC mixtures are spherical compared to elongated nematic tactoids for the biphasic regime of CNCs alone, a characteristic due to the small interfacial tension of liquid crystals of rodlike colloids in all-aqueous environments.^13,16,57^ Based on entropic and elastic energy considerations, the integration of hydrophobic, stiff BNNTs in a hydrophilic CNC matrix potentially modulates the overall alignment of mixed nanorods within the cholesteric shell, favoring the formation of spherical ChLC droplets in water. The mixing of larger-aspect-ratio BNNTs in the smaller-aspect-ratio CNC matrix likely promotes the alignment of nanorods, leading to a biphasic phase formation at a lower nanorod concentration, accompanied by the overall increase in entropy. This enables the potential templating of BNNTs within the cholesteric CNC layers of droplets, which gives rise to the elastic energy of droplets with an increasing droplet size to preserve the spherical packing of cholesteric layers. This type of mesogen organization satisfies the surface anchoring conditions where nanorods are aligned tangentially at the liquid crystal/water interface accompanied by the radial orientation of helical axis.^6^ This phenomenon was also observed previously for ChLC droplets of CNCs in a water/oil system, where the formation of concentric spherical layers was energetically preferred for micron-sized droplets with growing size as the elastic energy within the droplet scales as *KR* (i.e., *K* ≈ 10 pN and *R* is the average value of the Frank elastic constant for lyotropic liquid crystals and droplet radius, respectively).^6^ Specifically, the free energy of the formation of spherical ChLC droplets with concentric packing of cholesteric layers and an isotropic core contains the elastic and surface energy components, which scale as *E*_elastic_ = 8π*K*(*R* – *r*_i_) and *E*_surface_ = 4πσ*r*_i_^2^, respectively, where σ = 10^–7^ to 10^–6^ N m^–1^ is the interfacial tension between the isotropic and cholesteric phases of CNC dispersions and *r*_i_ is the isotropic core radius.^6^ Taking the droplet sizes (i.e., *R* = (40.0 ± 8.4)/2 μm and *r*_i_ = (11.6 ± 3.2)/2 μm) of BNNT/CNC mixtures at ϕ_BNNTs_ = 0.107 vol % as examples, the estimated elastic energy is in the range of 1–2 orders of magnitude larger than that of the surface energy component, suggesting that the elastic energy contributes mainly to the formation of spherical ChLC droplets. Additionally, free pectin in BNNT/CNC mixtures may contribute to the interfacial stabilization and liquid crystal configuration of ChLC shells in water due to its steric and amphiphilic nature that promotes hydrophobic and electrostatic interactions with stiff nanorods of CNCs and pectin-BNNTs at the liquid crystal/water interface. This nature is typical of amphiphilic polymers, such as poly­(vinyl alcohol), triblock copolymers, and polysaccharides, which are commonly used for interfacial stabilization of emulsions involving immiscible solvents (e.g., oil–water system) or water-immiscible thermotropic liquid crystal-in-water systems.^58,59^

Semiflexible pectin attains a coiled conformation in water, and its chain flexibility is dependent on various factors including the degree of esterification (DE), dissociation of carboxylic groups, and solvent environment.^60^ We estimated a DE value of ≈19% for our pectin sample based on the peak fitting of the ATR-FTIR spectrum (Figure S6).^46^ This lower DE value relates to a higher content of the −COOH group, the dissociation of which leads to increased electrostatic interactions of pectin. This can lead to increased pectin chain stiffness,^60^ which may promote the wrapping conformation on the BNNT surfaces during dispersion as well as interactions with stiff nanorods at the ChLC droplet interface. For control samples of CNC dispersions with pectin at concentrations (i.e., ϕ_CNC_ = 3.19 vol % and ϕ_pectin_ = 0.22 vol %) corresponding to those in typical BNNT/CNC mixtures, we observed a biphasic phase formation of nematic tactoids, although ϕ_CNC_ was smaller than ϕ_I,CNC_ ≈ 3.43 vol % for aqueous dispersions of CNCs alone (Figure S9a). We further examined CNC dispersions at ϕ_CNC_ = 3.19 ± 0.02 vol % with varying concentrations of pectin (Figure S10). We observed cholesteric-like tactoids of larger size at a lower pectin concentration of 0.10 vol % as compared to the nematic tactoids formed for CNC samples containing 0.22 vol % of pectin. At higher pectin concentrations of 0.49 and 0.94 vol %, the samples form even larger liquid crystal domains. These results suggest that pectin likely leads to the depletion-induced isotropic-liquid crystal transition of CNC dispersions, while the degree of depletion interactions is possibly dependent on the pectin concentration, in a manner similar to anionic and neutral polymers in modulating the CNC phase behavior, as reported previously.^61,62^ However, it is important to note that spherical ChLC shells did not form for CNC samples containing pectin without the addition of BNNTs, suggesting the key role of higher-aspect-ratio nanotubes in assisting the assembly of spherical ChLC droplets in BNNT/CNC mixtures (Figures S9a and S10). Here, we kept the free pectin concentration (i.e., corresponding to excess pectin in stock BNNT dispersions) constant in all of the BNNT/CNC mixture samples. Revealing the complex relationships of pectin features, such as concentration and molecular weight and functionalities, on the subsequent interfacial stabilization, depletion interactions, and tuning nanorod alignment within spherical ChLC droplets of BNNT/CNC mixtures is a topic worthy of future studies.

Next, we examined the effects of various factors including BNNT concentration and mixing time and condition on the structure and morphology of ChLC shells that are formed in aqueous BNNT/CNC mixtures (Figures 2, 3, S9, S11, and S12). We estimated the time for ChLC shell structures to reach the equilibrium state for BNNT/CNC mixtures at varying BNNT concentrations. For all BNNT/CNC mixtures, the samples form metastable nematic tactoids shortly after mixing (i.e., ≈30 min), which later coalesce into larger liquid crystal droplets with time and eventually transition to mostly spherical ChLC droplets with concentric cholesteric layers (Figure 2). Three other main types of morphologies such as uniaxial cholesteric tactoids-like structures,^13,14^ bipolar stripes,^6^ and transitional ellipsoidal concentric layers^6^ (i.e., corresponding to planar cholesteric pseudolayers in the center, while maintaining tangential nanorod orientation at the droplet periphery) were also identified in BNNT/CNC mixtures. Specifically, for BNNT/CNC mixtures at ϕ_BNNTs_ = 0.107 vol %, the population of spherical ChLC droplets with concentric cholesteric layers (i.e., ChLC droplet fraction) among all types of droplets formed increases with mixing time and reaches a plateau value of ≈43% at around 20 h, which is considered as the sample entering the equilibrium state (Figure 3 and Table 1). In general, the majority of these ChLC droplets are stable for up to roughly 24 h of mixing before the gradual change in characteristic structures of a Maltese cross and concentric cholesteric layers was observed, likely due to the unwinding of the cholesteric helix within droplet shells (Figures 2 and S11). This structural change is also accompanied by a decrease in the ChLC droplet fraction to ≈37% at 26 h of mixing for BNNT/CNC samples at ϕ_BNNTs_ = 0.107 vol % (Figure 3). In general, we observed that most ChLC shells maintain their characteristic morphology of concentric cholesteric layers up to the experimental time period of 5 days, demonstrating the relative stability of these liquid crystal droplets in all-aqueous environments (Figure S11). This dynamic, reconfigurable feature of the BNNT/CNC system can potentially enable the production of diverse ordered structures through on-demand crosslinking of liquid crystal droplets in future studies.

**Figure 2:**
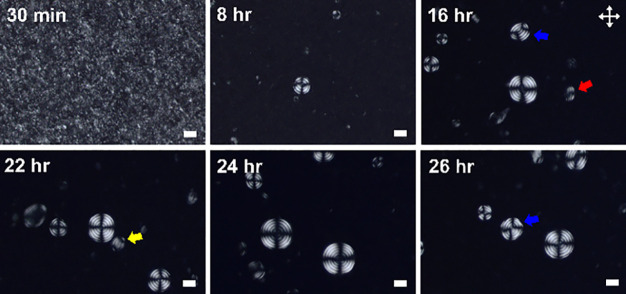
Representative POM images showing the time evolution of the BNNTs-assisted formation of spherical ChLC shells in aqueous dispersions of BNNT/CNC mixtures. Uniaxial cholesteric tactoid-like structures are indicated by red arrows, bipolar stripes by yellow arrows, and ellipsoidal concentric layers by blue arrows, respectively. The concentrations of nanorods are ϕ_CNC_ = 3.24 vol % and ϕ_BNNTs_ = 0.107 vol %, respectively. Scale bars are 20 μm.

**Figure 3:**
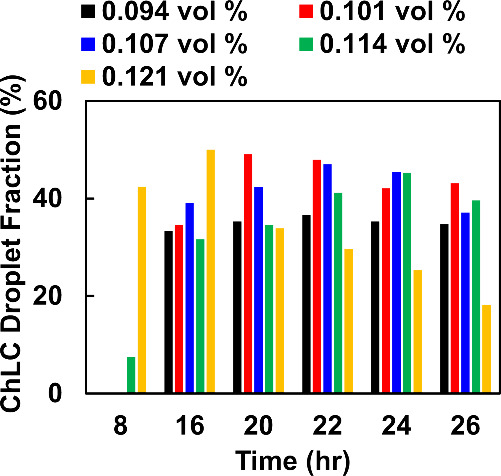
Time-resolved distribution of the population of spherical ChLC droplets with concentric cholesteric layers formed from BNNT/CNC mixtures at varying BNNT concentrations (i.e., ϕ_BNNTs_ = 0.094–0.121 vol %) in water. The CNC concentration in mixtures is ϕ_CNC_ = 3.23 ± 0.01 vol %. For each population, a minimum of 53 droplets was analyzed.

**Table 1:** Equilibration Time for the Fraction of Spherical ChLC Droplets with Concentric Cholesteric Layers Reaching a Plateau Value (i.e., Average ChLC Droplet Fraction) at Varying BNNT Concentrations

ϕ_BNNTs_ (vol %)	0.094	0.101	0.107	0.114	0.121
equilibration time (h)	16	20	20	22	16
average ChLC droplet fraction (%)	35 ± 1.1	46 ± 3.0	43 ± 3.7	43 ± 2.0	54^a^

a Peak value instead of the average ChLC droplet fraction.

For BNNT/CNC mixture samples at ϕ_BNNTs_ = 0.094–0.121 vol %, we found that the population of spherical ChLC droplets with concentric cholesteric layers and isotropic core (i.e., the radial point defect), corresponding to the ChLC droplet fraction, is dependent on both BNNT concentration and mixing time (i.e., 8–26 h) (Figure 3). In comparison, BNNT/CNC mixtures with lower BNNT concentrations at ϕ_BNNTs_ = 0.013–0.081 vol % displayed nematic tactoids without the formation of spherical ChLC shells even after 24 h of mixing (Figure S12). When ϕ_BNNTs_ increased from 0.101 to 0.121 vol %, the equilibration time for the fraction of ChLC droplets to reach a plateau value decreased from roughly 20 to 16 h of mixing. Except for BNNT/CNC mixtures at higher ϕ_BNNTs_ of 0.114 and 0.121 vol %, we did not observe the formation of spherical ChLC shells with 8 h of mixing. Despite forming spherical ChLC shells at a shorter time (i.e., within 8 h), BNNT/CNC mixtures at a higher ϕ_BNNTs_ of 0.121 vol % displayed a narrow time range for maintaining the optimum value of ChLC droplet fraction, indicating the important role of BNNT concentration in tuning the droplet structures. Although BNNT/CNC mixtures at ϕ_BNNTs_ = 0.094 vol % entered the equilibrium state approximately after 16 h of mixing, the fraction of ChLC droplets remained the lowest at ≈35%. This is likely due to an insufficient amount of BNNTs in assisting the formation of spherical ChLC shells, among other competing structures, by interacting with the CNC matrix. Additionally, control BNNT/CNC mixtures at a higher concentration of ϕ_BNNTs_ = 0.121 vol %, which were kept stationary, did not form ChLC shells (Figure S9b,c). This suggests that mixing potentially promotes the interactions between nanorods and pectin molecules as well as the coalescence of smaller liquid crystal tactoids to facilitate the formation of ChLC shells, while maintaining uniform dispersions of nanorods during the phase transition by minimizing nanorod sedimentation and bulk phase separation to occur.^16,17^ Tuning the equilibrium state as well as the sizes, structures, and population of spherical ChLC droplets in BNNT/CNC mixtures by further modulating nanorod concentrations, mixing time, and conditions (e.g., orbital radius, rotation rate), surface coatings of nanotubes, and interfacial stabilization is a topic worthy of future studies.

We further examined the effects of BNNT concentration and mixing time on the morphology of nanorod confinement in spherical droplets by analyzing the droplet sizes (i.e., droplet and core diameters) and pitch of ChLC shells (Figure 4). The spherical confinement of concentric cholesteric layers with tangential anchoring of the director ***n*** at the droplet interface, corresponding to the tangential alignment of mesogenic molecules at the liquid crystal/solvent interface, is required to acquire a radial point defect at equilibrium to avoid the high elastic energy of distortions at the droplet center.^6,63^ This is reflected by the term −8π*Kr*_i_ in the elastic energy component, which favors the acquisition of the isotropic core. This topological defect is shown macroscopically as an isotropic phase at the droplet core, which is on the micron scale for the previously reported ChLC droplets formed from lyotropic liquid crystal of CNCs.^6^ Here, by increasing the BNNT concentration from 0.094 to 0.121 vol %, we observed an increase of the overall diameter of ChLC droplets from 25.1 ± 6.6 to 42.5 ± 6.9 μm, whereas the isotropic cores increased from 6.1 ± 1.6 to 13.7 ± 3.0 μm (Figure 4a). This corresponds to an ≈53% increase in the ChLC shell thickness (i.e., roughly from 19 to 29 μm), suggesting that BNNTs likely phase-separates primarily into liquid crystals shells as opposed to the required isotropic cores (i.e., topological defect). This observed growth of concentric cholesteric layers of droplets with increasing BNNT concentration in BNNT/CNC mixtures follows the entropic and elastic energy considerations of spherical packing of cholesteric layers as discussed above. However, the potential distribution of BNNTs into the ChLC shells primarily in this mixed nanorod system of BNNT/CNC may be further rationalized by future studies. Meanwhile, the change in corresponding cholesteric pitch is less significant, which increased by ≈12% from 7.7 ± 0.6 to 8.6 ± 0.7 μm, suggesting a slight difference in the packing distance of cholesteric concentric layers as droplets grow (Figure 4a). These results show that the addition of a small amount of BNNTs promotes the growth of ChLC shells in BNNT/CNC mixtures by sensitively tuning the droplet size without drastically changing the pitch. Taking the BNNT/CNC mixture at ϕ_BNNTs_ = 0.121 vol % as an example, we further observed that the overall changes in sizes of ChLC droplets (i.e., from 34.5 ± 6.3 to 41.2 ± 9.0 μm), isotropic cores (i.e., from 8.8 ± 2.1 to 14.7 ± 4.2 μm), and pitch (i.e., from 8.6 ± 0.7 to 8.8 ± 0.7 μm) with time are small across the experimental time period (i.e., 8–26 h) (Figure 4b). This suggests that once ChLC droplets are formed at a given BNNT concentration, these structures remain relatively stable within the time period tested, especially maintaining a roughly constant pitch size.

**Figure 4:**
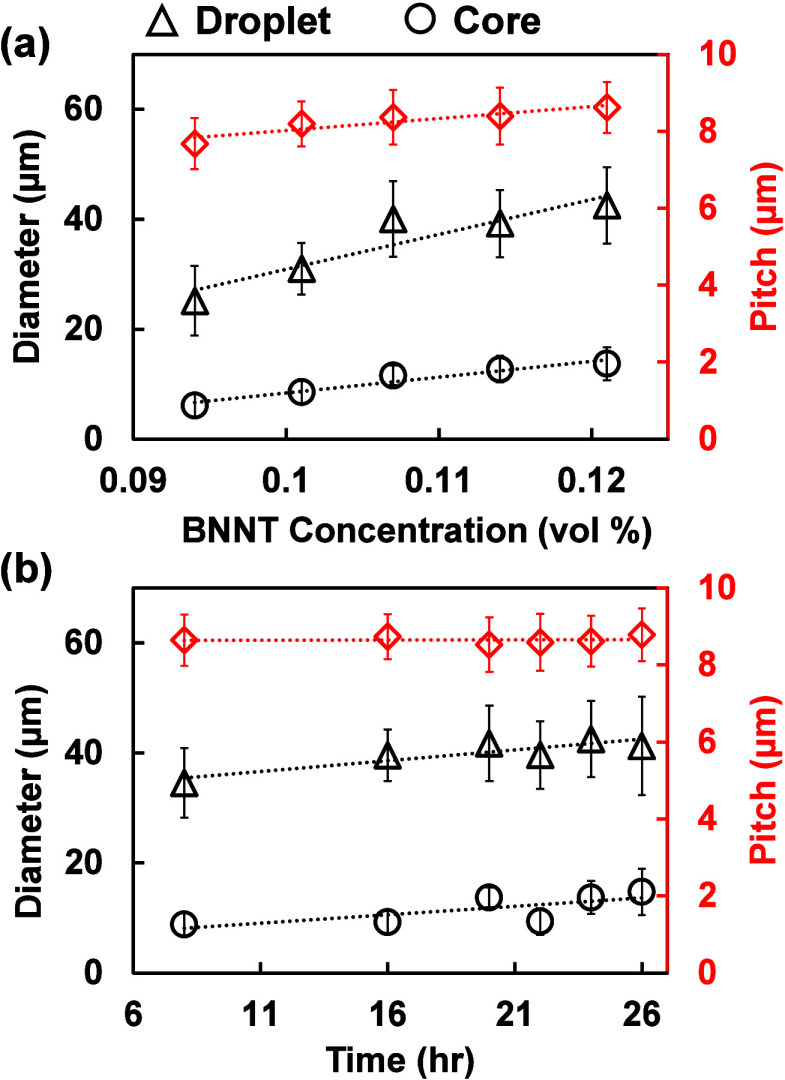
Structural evolution of spherical ChLC droplets with concentric cholesteric layers formed from BNNT/CNC mixtures in water. (a) Variations in the diameters of ChLC droplets (black triangles) and isotropic cores (black circles) and cholesteric pitch (red diamonds) as a function of the BNNT concentration. Samples were characterized after mixing for 24 h. (b) Size variations of spherical ChLC droplets as a function of time for a BNNT/CNC mixture containing ϕ_CNC_ = 3.22 vol % and ϕ_BNNTs_ = 0.121 vol %, respectively. For each experimental point, a minimum of 51 droplets were analyzed.

## Conclusions

Spontaneous assembly of lyotropic stiff nanorods into spherical ChLC droplets with an isotropic core in water is an interesting topic for both basic research and technological applications. We demonstrated that the mixing of hydrophobic BNNTs in aqueous dispersions of hydrophilic CNCs assisted the formation of spherical ChLC droplets (i.e., liquid crystal-in-water emulsion) of the micrometer scale, rather than forming typical elongated tactoids in water-in-water emulsions. Specifically, adding small amounts of larger-aspect-ratio pectin-BNNTs (i.e., ϕ_BNNTs_ = 0.094–0.121 vol %) that were tested in this work, we observed the formation of ChLC shells in the CNC matrix of ϕ_CNC_ = 3.20 ± 0.04 vol %, which was originally isotropic. This is consistent with the Onsager theory, where critical concentrations for phase transition correspond to the inverse of the aspect ratio of lyotropic rods. Additionally, the thickness of ChLC shells grows by ≈53% (i.e., roughly from 19 to 29 μm) with respect to a slight increase in the BNNT concentration, suggesting that BNNTs likely phase separate primarily into liquid crystal shells as opposed to the isotropic cores. Our results also suggest a synergistic liquid crystal phase behavior of mixed mesogenic nanorods of BNNTs and CNCs in water, opening possibilities for developing ordered structures with integrated nanomaterial properties and applications, such as photonics, optical films, and platforms for encapsulation and material templating. Our spherical ChLC droplet system was formed in all-aqueous environments spontaneously, allowing potential incorporation of water-soluble molecules, such as biomolecules and nutrients, within the isotropic core that is protected by a mechanically robust, optically active nanorod-reinforced shell. This paves the way for further development of all-aqueous smart delivery systems and photonic structures. Future work in our lab will focus on revealing the structure–property relationships in forming spherical ChLC droplets in lyotropic mixed nanorod systems by further exploring various aspects of nanorods including purity, a broader concentration range, and surface chemistry of nanorods as well as on achieving mechanically robust ChLC droplets for advanced manufacturing of optical films, photonics, and smart delivery system applications.

## Supplementary Material



## Notes

Spectroscopic characterization of pectin, CNC, and BNNT samples, TEM and length distribution characterization of CNC samples, optical microscopy of CNC dispersions and BNNT/CNC mixtures, ATR-FTIR spectra of pectin, and supplementary tables (PDF)
