# Supplementary material for: Boron Nitride Nanotubes Assist the Self-Assembly of Spherical Cholesteric Liquid Crystal Shells of Cellulose Nanocrystals in Water
Source: Langmuir. 2026 May 4;42(19):13469–77. doi: 10.1021/acs.langmuir.6c00394 (PMC13192314; doi:10.1021/acs.langmuir.6c00394)
Supplement: Supplementary file 1 [file la6c00394_si_001.pdf]

Supporting Information

Boron Nitride Nanotubes Assist the Self-Assembly of Spherical Cholesteric Liquid Crystal Shells of Cellulose Nanocrystals in Water

Tanner L. Larson,[1,2] *Brandon J. Heppe,*[1] *Benjamin S. Flavel,*[2] *Ralph Krupke,*[2,3,4] *Geyou Ao*[1,2]*

[1]Department of Chemical and Biomedical Engineering, Washkewicz College of Engineering, Cleveland State University, 2121 Euclid Avenue, Cleveland, OH 44115, United States

[2]Institute of Nanotechnology, Karlsruhe Institute of Technology, 76131 Karlsruhe, Germany

[3]Department of Materials Science, Technical University of Darmstadt, 64287 Darmstadt, Germany

[4]Institute of Quantum Materials and Technologies, Karlsruhe Institute of Technology, 76131 Karlsruhe, Germany

Corresponding Author

*Email address: g.ao@csuohio.edu

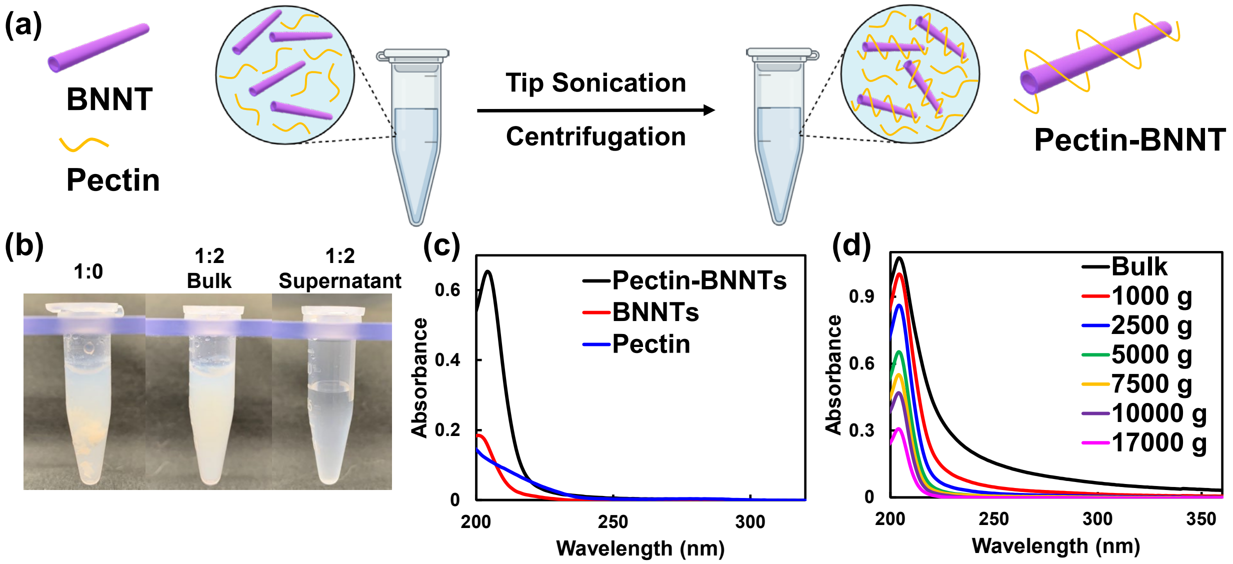

Figure S1. Aqueous dispersions of pectin-coated BNNT complexes. (a) Scheme for producing stable pectin-BNNT dispersions in water. (b) Photographs of samples with BNNTs:pectin mass ratios of 1:0 and 1:2 before (i.e., bulk) and after (i.e., supernatant) centrifugation at 5000 g. (c) Representative absorbance spectra of pectin only, BNNTs only, and stock pectin-BNNT supernatant samples after 5000 g centrifugation that are prepared at the same initial concentrations of each component. (d) Representative absorbance spectra of bulk and supernatant dispersions with BNNTs:pectin = 1:2 mass ratio collected after varying centrifugation at 1000–17000 g. The starting BNNT concentration is 1 mg/mL.

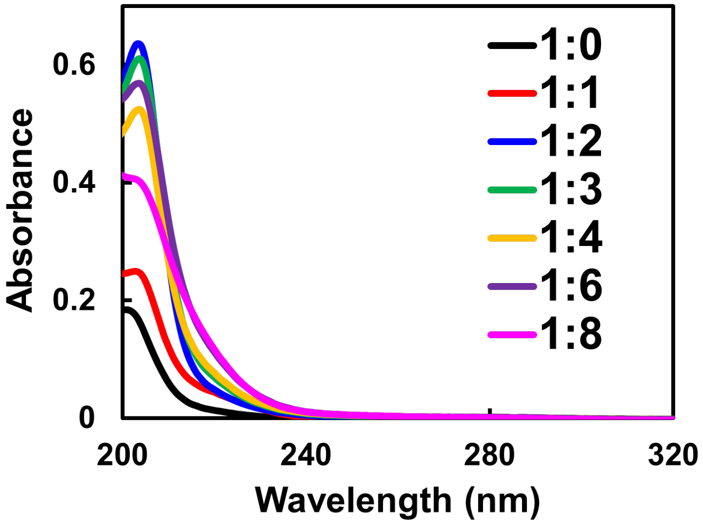

Figure S2. Representative absorbance spectra of supernatant dispersions of pectin-BNNTs at different BNNTs:pectin mass ratios of 1:n, where n = 0, 1, 2, 3, 4, 6, and 8, respectively. The starting BNNT concentration is 1 mg/mL. Supernatant dispersions were collected after centrifugation at 17000 g.

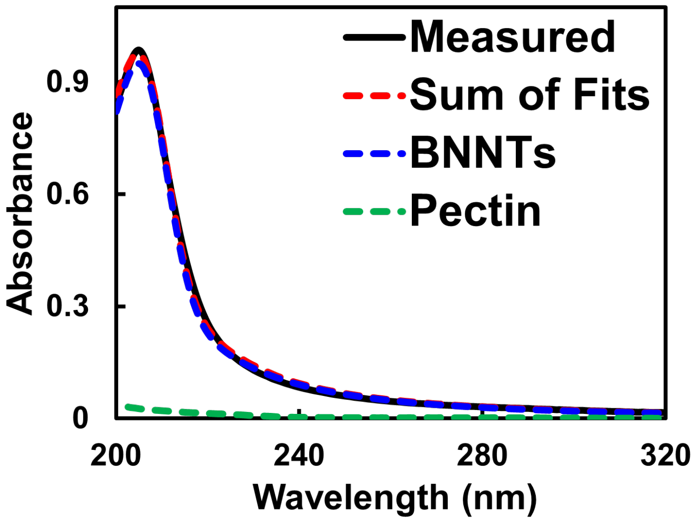

Figure S3. Absorbance spectra of supernatant dispersions of pectin-BNNTs in water and the corresponding peak fitting through multiple linear regression of absorption peaks of each component (i.e., free pectin and BNNTs, respectively) to obtain a global fitting of the measured absorbance spectrum. The starting BNNT concentration is 4 mg/mL.

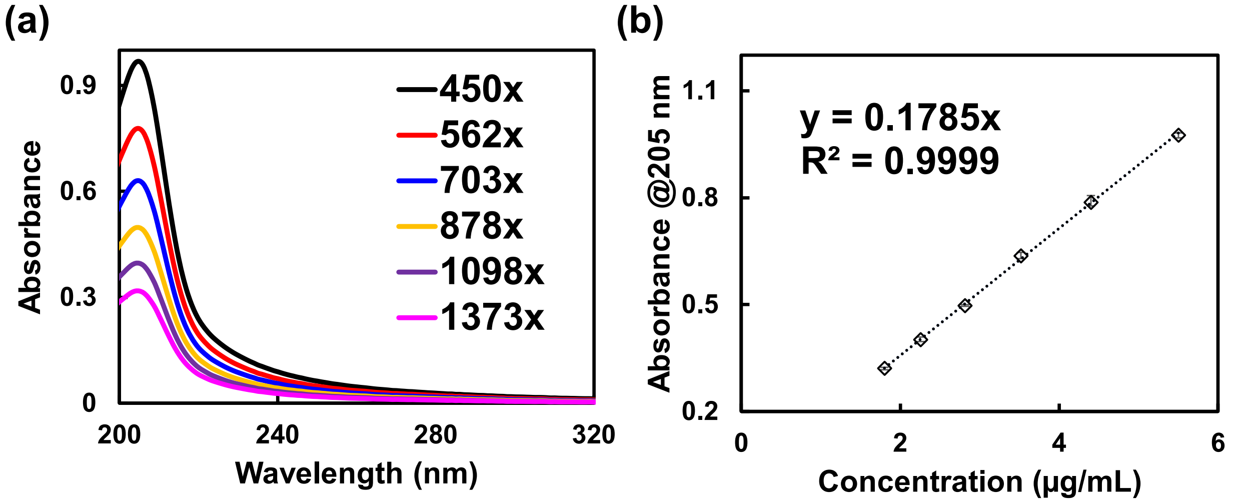

Figure S4. Determining the extinction coefficient of pectin-BNNT complexes at 205 nm in water. (a) Representative absorbance spectra of pectin-BNNTs obtained from peak fitting at different dilution factors ranging from 450× to 1373× in water. (b) The calibration curve of pectin-BNNT absorbance values at 205 nm as a function of nanotube concentration. The extinction coefficient is determined to be 178.54 mL mg^{-1} cm^{-1} at 205 nm. Error bars were generated from the standard deviation of three repeats.

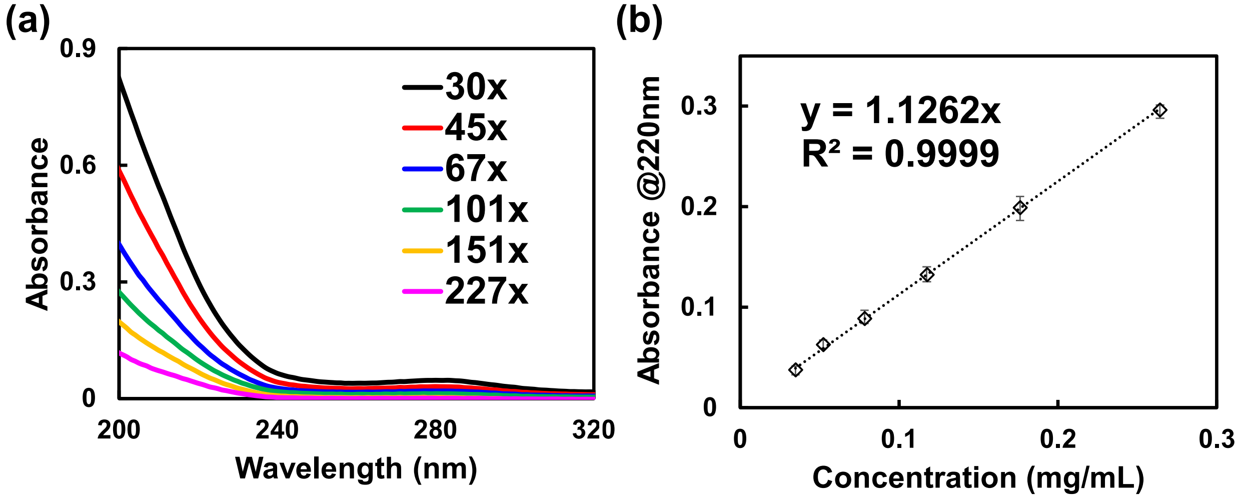

Figure S5. Determining the extinction coefficient of pectin only solutions at 220 nm in water. (a) Representative absorbance spectra of pectin solution at different dilution factors ranging from 30× to 227× in water. (b) The calibration curve of pectin absorbance values at 220 nm as a function of pectin concentration. The extinction coefficient is determined to be 1.13 mL mg^{-1} cm^{-1} at 220 nm. Error bars were generated from the standard deviation of three repeats. The stock solution of 8 mg/mL pectin was prepared by following the same method used for pectin-BNNT dispersions.

Conversion of BNNT concentrations from mass % to vol %

The following equation was used to convert BNNTs mass % (w_{BNNTs}) to vol % (i.e., ϕ_{BNNTs}) for BNNT/CNC mixtures in water.

$$\phi_{BNNTs} = \frac{m_{BNNTs}/\rho_{BNNTs}}{m_{BNNTs}/\rho_{BNNTs} + m_{CNC}/\rho_{CNC} + m_{pectin}/\rho_{pectin} + m_{solvent}/\rho_{solvent}}$$

Here, m denotes mass and ρ denotes density.

Table S1. Conversions of BNNTs mass % (i.e., w_{BNNTs}) to vol % (i.e., ϕ_{BNNTs}) for representative BNNT/CNC mixture samples in water. CNC concentrations in mixtures were kept constant at 5.14 ± 0.06 mass % (i.e., ϕ_{CNC} = 3.20 ± 0.04 vol %).

w_{BNNTs} (mass %)	ϕ_{BNNTs} (vol %)	Total nanorod concentration $\phi_{BNNTs+CNC}$ (vol %)
0.020	0.013	3.20
0.080	0.054	3.18
0.118	0.081	3.27
0.138	0.094	3.32
0.148	0.101	3.31
0.157	0.107	3.35
0.167	0.114	3.35
0.177	0.121	3.35

Table S2. Zeta potential and pH measurements of various samples in water.

**Samples**	**Zeta potential (mV)**	**pH**
Pectin only	−37 ± 0.81	4.28 ± 0.02
CNCs only	−66 ± 2.55	5.97 ± 0.06
Pectin-BNNTs	−40 ± 0.78	4.86 ± 0.13

*Pectin only sample was prepared by following the same method used for pectin-BNNT dispersions.

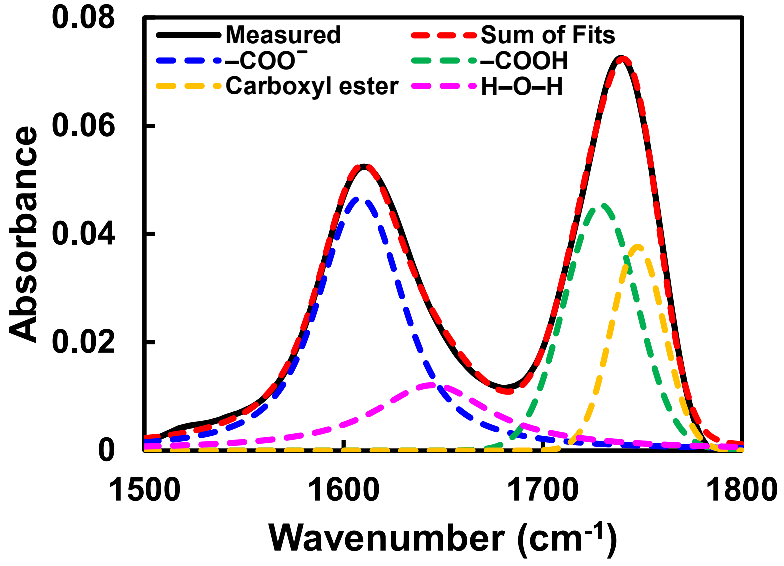

Figure S6. ATR-FTIR spectrum of pectin and the corresponding Voigt curve fitting. The blue, green, and yellow dotted lines correspond to the absorption bands of asymmetric stretching vibration of $–COO^{–}$ as well as C=O stretching vibrations of –COOH and carboxyl ester groups, respectively. The pink dotted line corresponds to the absorption band of residual H–O–H bending from adsorbed water.[1]

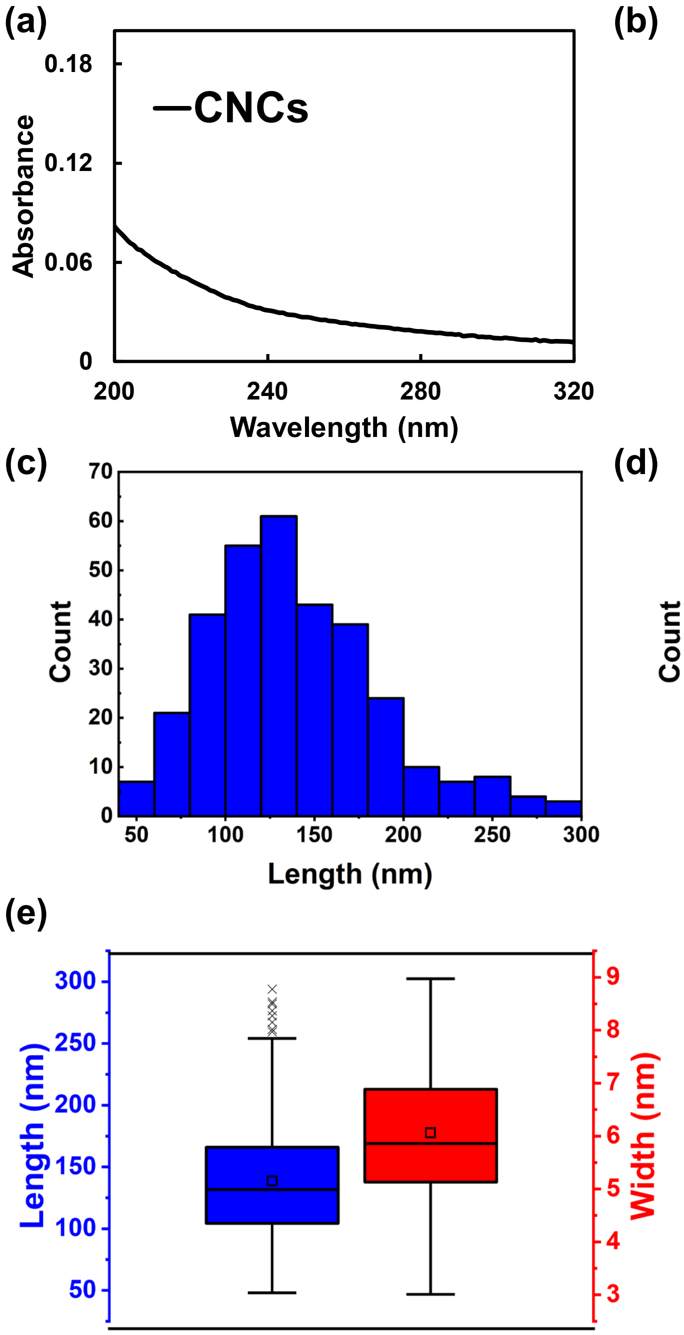

Figure S7. Characterization of CNCs. (a) Representative absorbance spectrum of CNC dispersion. (b) Representative TEM image of CNCs (scale bar of 200 nm). Histograms for the distributions of (c) length and (d) width of CNCs obtained from imaging of 324 and 261 nanorods, respectively, and corresponding (e) box-and-whisker plots. The boxes represent the 25–75 percentile data and median values, while the average is indicated by the square symbol. The whiskers represent minimum and maximum values, while the outliers are indicated by the cross symbol.

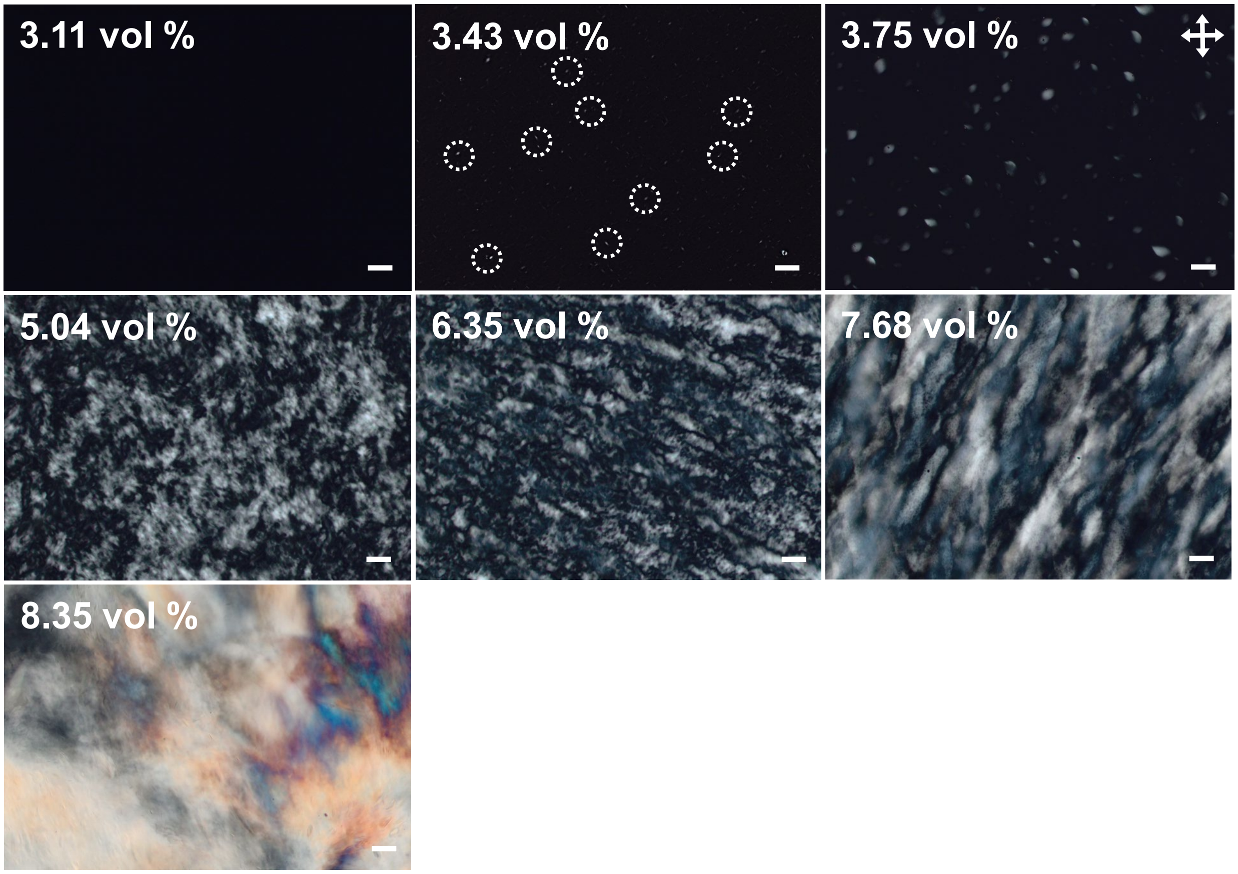

Figure S8. Cross-polarized optical light microscopy (POM) images of aqueous dispersions of CNCs (without BNNTs and free pectin) at varying ϕ_{CNC} = 3.11–8.35 vol % (i.e., 5.00–13.0 mass %). White circles in the image of 3.43 vol % sample indicate tactoids. Scale bars are 20 µm.

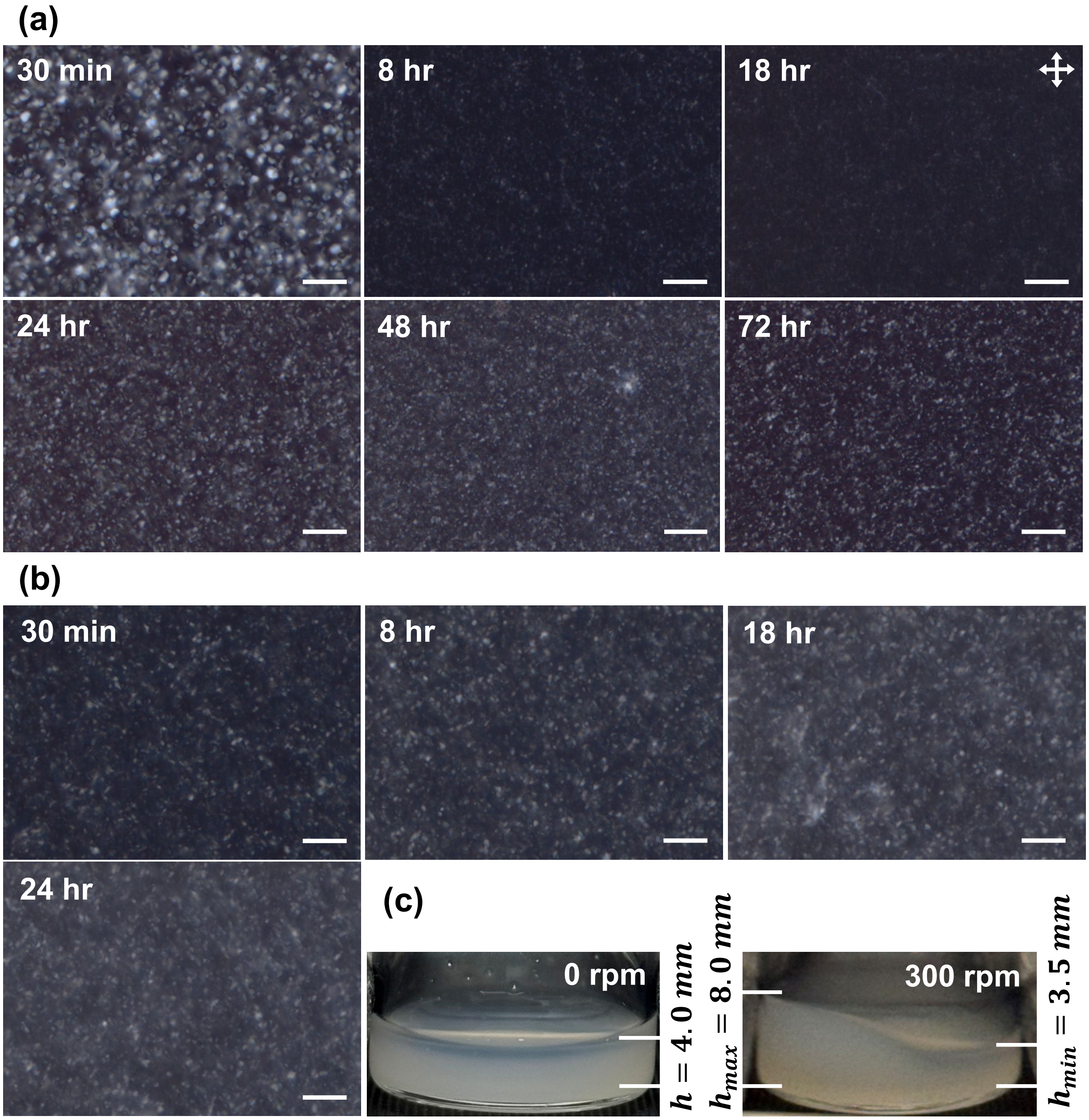

Figure S9. Control samples. (a) Representative POM images showing the structural evolution of CNC dispersions with pectin (without BNNTs) at $\phi_{CNC} = 3.19$ vol % at varying mixing time. A final concentration of 0.35 mass % (i.e., 0.22 vol %) pectin was added, which corresponds to the amount of free pectin in a typical BNNT/CNC mixture. (b) Representative POM images of a BNNT/CNC mixture at $\phi_{CNC} = 3.16$ vol %, $\phi_{BNNTs} = 0.121$ vol %, and excess free pectin of $\phi_{pectin} = 0.16$ vol % (i.e., 2.70 mg/mL), that is kept stationary. (c) Photographs of vials at stationary and under rotation at 300 rpm on an orbital shaker with the average values of sample thickness (h) indicated. h values were measured using Image J. Scale bars are 20 µm.

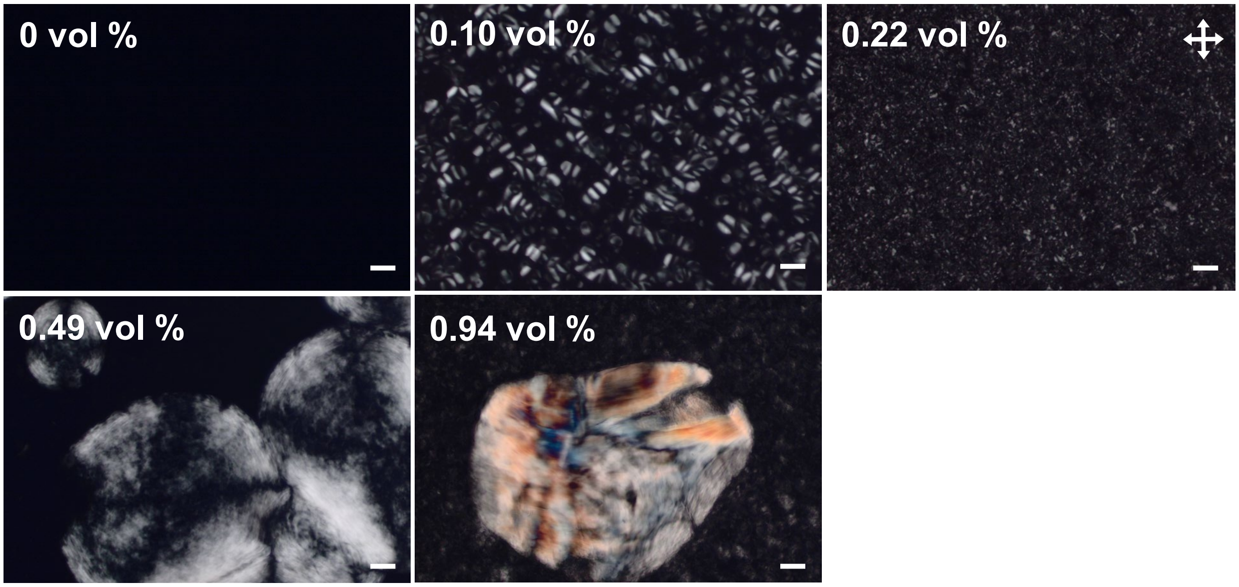

Figure S10. Representative POM images showing the liquid crystal phase behavior of aqueous CNC dispersions (without BNNTs) at ϕ_{CNC} = 3.19 ± 0.02 vol % with varying concentrations of pectin (i.e., 0−0.94 vol %) after 24-hour mixing. Scale bars are 20 μm.

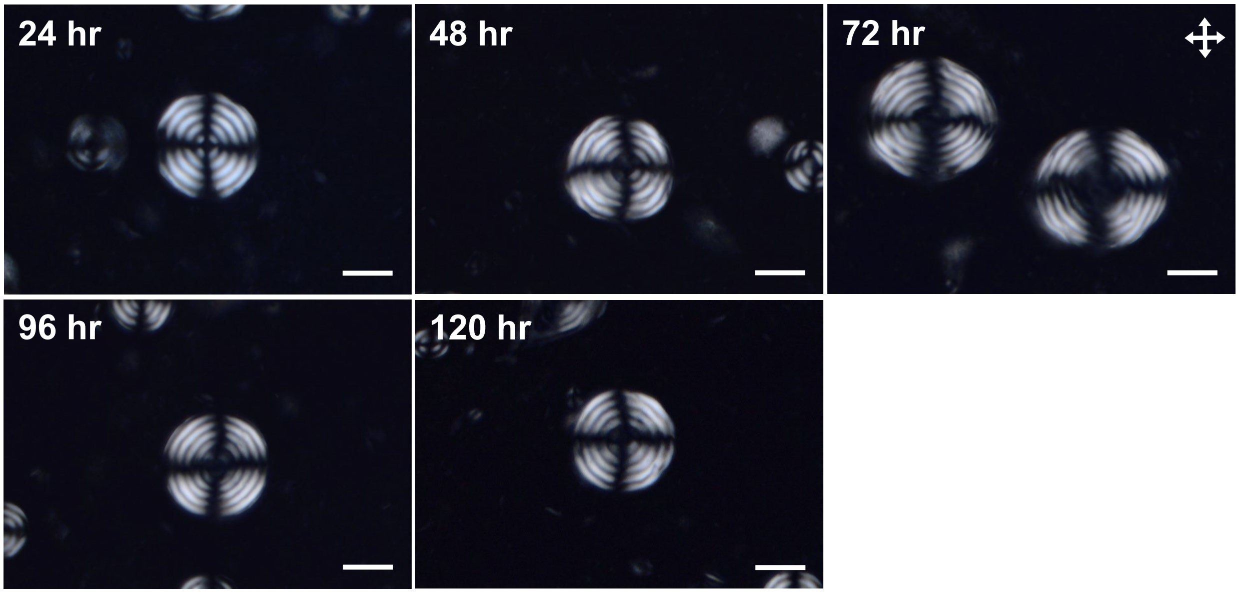

Figure S11. The stability of ChLC shells obtained from BNNT/CNC mixtures as a function of time. Representative POM images of ChLC shells in a mixture sample containing ϕ_{CNC} = 3.21 vol %, ϕ_{BNNTs} = 0.107 vol %, and excess ϕ_{pectin} = 0.21 vol % (i.e., 3.54 mg/mL). Scale bars are 20 μm.

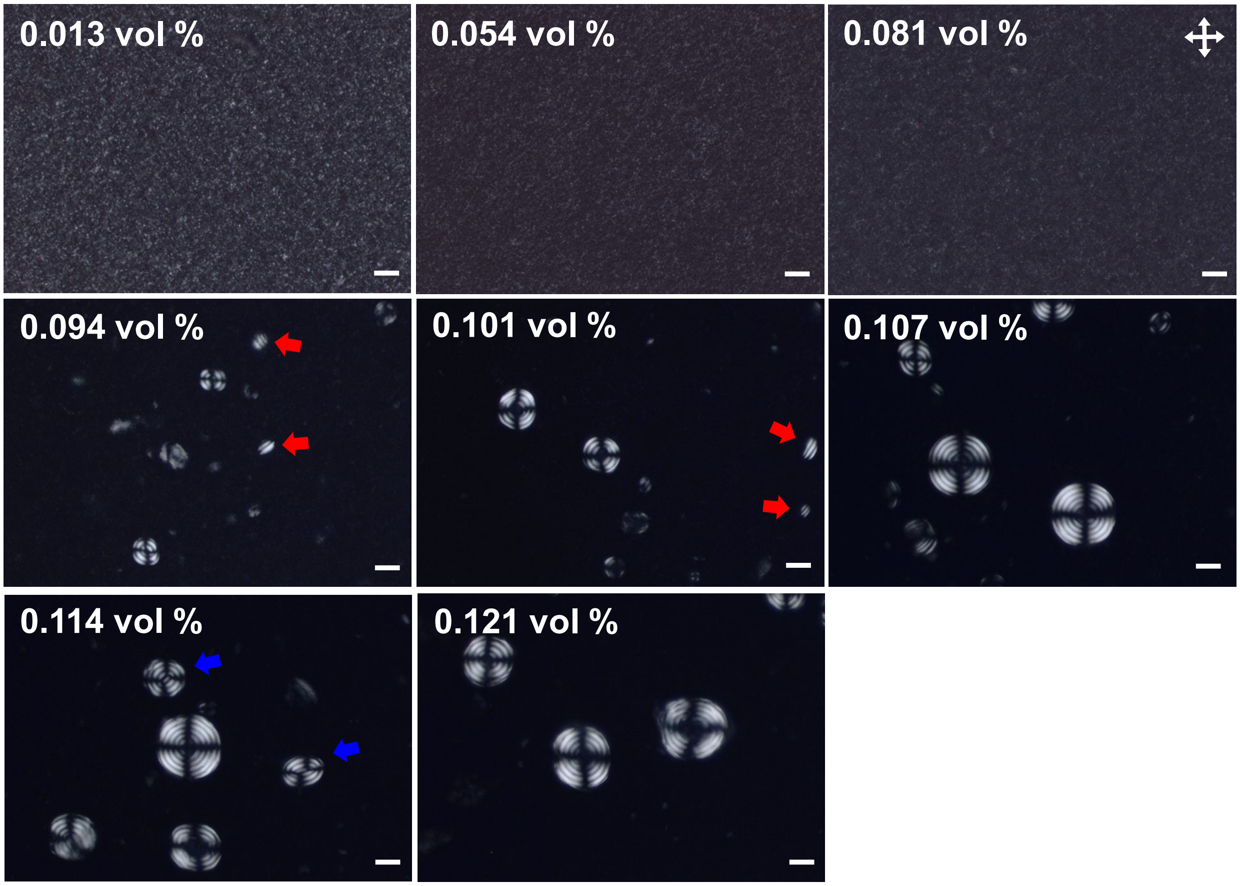

Figure S12. Representative POM images of BNNT/CNC mixtures showing the structural evolution of ChLC shells as a function of BNNT concentration with 24-hour mixing. Uniaxial cholesteric tactoids-like structures are indicated by red arrows and ellipsoidal concentric layers by blue arrows, respectively. Samples have varying ϕ_{BNNTs} = 0.013–0.121 vol %, while maintaining constant values of ϕ_{CNC} = 3.20 ± 0.04 vol % and ϕ_{pectin} = 0.18 ± 0.03 vol % (i.e., 3.03 ± 0.43 mg/mL). Scale bars are 20 μm.

REFERENCES

(1) Yu, CC.; Chiang, KY.; Okuno, M.; Seki, T.; Ohto, T.; Yu, X.; Korepanov, V.; Hamaguchi, H.; Bonn, M.; Hunger, J.; Nagata, Y. Vibrational Couplings and Energy Transfer Pathways of Water's Bending Mode. *Nat. Commun.* **2020**, *11*, 5977. DOI: 10.1038/s41467-020-19759-w.
